# Respiratory variation in peripheral arterial blood flow peak velocity to predict fluid responsiveness in mechanically ventilated patients: a systematic review and meta-analysis

**DOI:** 10.1186/s12871-018-0635-0

**Published:** 2018-11-13

**Authors:** Bo Yao, Jian-yu Liu, Yun-bo Sun

**Affiliations:** 1 The department of critical care medicine in the affiliated hospital of Qingdao university, Jiangsu road 16, Qingdao city, 26600 China; 2 The institute of organ transplantation in Qingdao university, Jiangsu road 16, Qingdao city, 26600 China

**Keywords:** Fluid loading, Fluid responsiveness, Publication bias, Peripheral arterial blood flow peak velocity

## Abstract

**Background:**

Fluid overloading is detrimental to organ function and results in a poor prognosis. It is necessary to evaluate fluid responsiveness before fluid loading. We performed a systematic meta-analysis to evaluate the diagnostic value of the respiratory variation in peripheral arterial blood flow peak velocity (△Vpeak PA) in predicting fluid responsiveness in mechanically ventilated patients.

**Methods:**

PubMed, Embase and The Cochrane Library databases were searched for studies that used △Vpeak PA to predict fluid responsiveness in mechanically ventilated patients. We calculated the pooled values of sensitivity, specificity and the area of the summary receiver operating characteristic curve by Meta-Disc 14.0 software.

**Results:**

Nine studies with a total of 402 patients were included. Two low quality studies were deleted in further analysis. Moreover, because of different locations of peripheral artery, the rest included studies were divided into brachial site group and carotid site group for meta-analysis individually. The pooled sensitivity, specificity and area under curve were 0.85 (95% confidence interval (CI) 0.77–0.92), 0.86 (95% CI 0.77–0.92) and 0.9268 in carotid site group. The pooled sensitivity, specificity and area under curve were 0.72 (95% CI 0.60–0.81), 0.85 (95% CI 0.74–0.93) and 0.8587 in brachial site group.

**Conclusions:**

△Vpeak of carotid and brachial artery had a diagnostic value in predicting fluid responsiveness respectively. Moreover, △Vpeak of carotid artery had more value than brachial artery in predicting fluid responsiveness. However, there was some clinical heterogeneity; therefore, further studies are needed to confirm diagnostic accuracy.

## Background

Fluid resuscitation is the basic therapy for shock, but fluid overloading is detrimental to organ function and results in a poor prognosis [1]. In the condition of shock, the purpose of fluid loading is to increase the cardiac output to alleviate the hypo-perfusion. Those whose cardiac output increases significantly after fluid loading are called fluid responders. However, fluid responders made up only half of a population of critically ill patients [2]. Therefore, it is necessary to evaluate fluid responsiveness before fluid loading.

The parameters for predicting fluid responsiveness include static and dynamic indicators. Static indicators (central venous pressure or pulmonary artery wedge pressure) are not recommended as good diagnostic indexes [3, 4]. Some dynamic indicators, such as stroke volume variation (SVV) and pulse pressure variation (PPV), have better diagnostic value for predicting fluid responsiveness [5]. However, it is necessary for physicians to perform invasive vessel puncturing to monitor PPV or SVV. Physicians with professional ultrasound diagnostic skills can also obtain SVV by ultrasound. However, sometimes it can be difficult to obtain a clear transthoracic echocardiographic image to measure SVV. Moreover, when surgeons are conducting chest or abdominal operations, the anesthetists may not be able to obtain the transthoracic echocardiographic image.

Blood flowing in the vessels generates blood pressure and cardiac output; thus, there may be some relationship between blood flow velocity and blood pressure or cardiac output. It has been proved that peripheral blood flow velocity (such as carotid blood flow velocity) is quite relative to cardiac output (*r* = 0.8, *P* < 0.01) [6]. Measuring peripheral blood flow velocity is also easier than measuring stroke volume by transthoracic echocardiography. Moreover, a peripheral artery is shallow, so it is easy to obtain high-quality ultrasound images. Some previous studies have proved the value of peripheral arterial blood flow peak velocity (△Vpeak PA) to predict fluid responsiveness [6–13], but the study sample sizes were small, and the results were not always consistent. Therefore, we performed a meta-analysis to further evaluate the accuracy value of △Vpeak PA in predicting fluid responsiveness in mechanically ventilated patients.

## Materials and methods

### Search strategy

Databases for PubMed, Embase and The Cochrane Library were searched for relevant publications up to June 2017 with diagnostic trials about the value of △Vpeak PA in predicting fluid responsiveness in mechanically ventilated patients. The search terms were “carotid OR femoral OR brachial OR radial artery” and “fluid OR volume responsiveness”. No language restriction was applied. The search strategy was performed independently by two investigators (Y.B. and L.J.Y). If a discrepancy existed between the two authors, it was resolved by discussion.

### Study selection

In the first step, duplicate articles were deleted from the primary screening articles. In the second step, non-clinical studies were excluded. In the third step, by screening titles and abstracts, articles were selected if the studies were about the value of △Vpeak PA to predict fluid responsiveness. Studies then were included if all the following criteria were fulfilled: (1) The population consisted of patients who were mechanically ventilated. (2) The results of study included the sensitivity, specificity, area under the receiver operating characteristic curve and cut-off value. (3) Only studies published as full-text articles were included. Study selection was performed independently by two investigators (Y.B. and L.J.Y). If a discrepancy existed between the two authors, it was resolved by discussion.

### Data extraction and quality assessment

The following data were extracted from each included study: the characteristics of study (year of publication, study design), population (primary disease, sample size, inclusion criteria and exclusion criteria), mechanical ventilation parameters, location of peripheral artery, methods used to perform fluid responsiveness, and diagnostic values (sensitivity, specificity, an area under the receiver operating characteristic curve and cut-off value). Study quality was assessed by the Quality Assessment of Diagnostic Accuracy Studies 2 (QUADAS-2) tool [14]. Data extraction and quality assessment were performed independently by two investigators (Y.B. and L.J.Y). If a discrepancy existed between the two authors, it was resolved by discussion.

### Statistical analysis

Meta-Disc (version 1.4) software was used for data analysis. Statistical heterogeneity caused by the threshold effect was assessed by calculating the Spearman correlation coefficient of sensitivity and 1-specificity logarithmic. If *P* < 0.05, there was no statistical heterogeneity caused by the threshold effect. Statistical heterogeneity caused by the non-threshold effect between studies was assessed by using the I^2^ test. I^2^ ≥ 50% was considered to be statistically significant heterogeneity. I^2^ < 25% meant non-significant heterogeneity. A fixed-effect model was used for the meta-analysis if statistical heterogeneity did not exist. A random-effect model was used for the meta-analysis if statistical heterogeneity existed. The overall pooling of sensitivity, specificity, positive likelihood ratio, negative likelihood ratio, and diagnostic odds ratio were calculated by using the relative model. A summary receiver operating characteristic curve was constructed, and an area under the receiver operating characteristic curve and Q value was calculated. The Harbord test was applied to determine the presence of publication bias using the Stata (version 14.0) software.

## Results

**Fig. 1:**
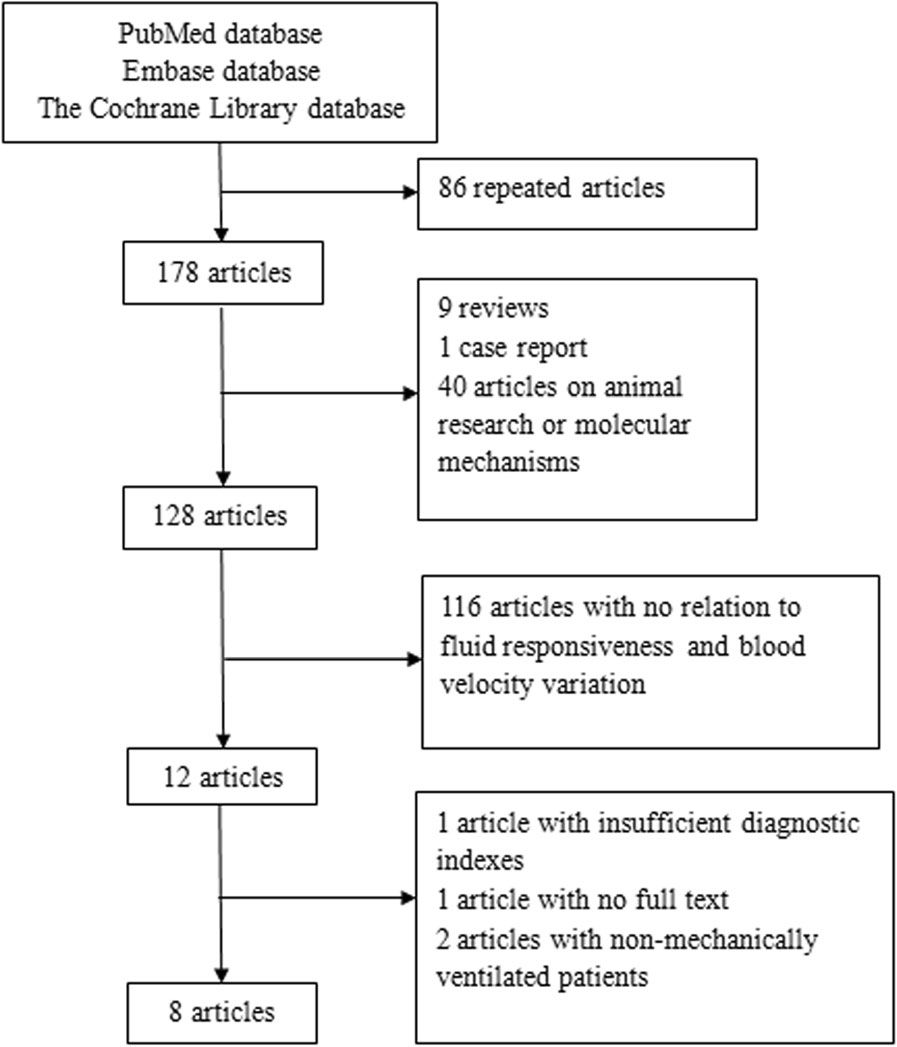
Flow chart of study selection and inclusion

The process of study selection and inclusion is illustrated in Fig. 1. Finally, eight articles were included [6–13]. Moreover, the LU N [13] study can be regarded as two studies because peripheral arteries from two different locations (carotid and brachial artery) were studied. A total of 402 patients were enrolled in the nine studies. Among them, 211 patients (52.5%) were responders to a fluid challenge. Characteristics of included studies were shown in Table 1.

**Table 1:** Characteristics of included studies

Author	Year	Sample size	Population	Breath type	Location of peripheral artery	Criterion of fluid responsiveness
Roehrig C [6]	2017	33	Cardiac surgery	PSV or SIMV mode, spontaneous breath is allowed VT = 6-8 ml/kg, PEEP = 5cmH2O	Carotid artery	Passive leg raising test
Brennan JM [7]	2007	30	Not mentioned in detail	VC mode VT ≥ 8 ml/kg, PEEP = 5–10 mmHg	Brachial artery	PPV ≥ 13%
Monge García MI [8]	2009	38	Patients with acute circulatory failure	VC mode VT =9 ml/kg, PEEP = 6 cmH2O	Brachial artery	Classic fluid responsiveness test
Yin WH [9]	2013	46	Abdominal, craniocerebral and orthopaedics surgery	VC mode VT = 8–12 ml/kg, PEEP = 5–10 mmHg	Carotid artery	Classic fluid responsiveness test
Song Y [10]	2014	40	Coronary artery surgery	VC mode VT =8 ml/kg, PEEP = 5 cmH2O	Carotid artery	Classic fluid responsiveness test
Ibarra-Estrada MÁ [11]	2015	59	Septic shock	VC mode VT = 6 ml/kg, PEEP = 6 cmH2O	Carotid artery	Classic fluid responsiveness test
ZHU W [12]	2016	58	Septic shock	PSV mode VT is not mentioned	Brachial artery	Classic fluid responsiveness test
LU N [13]	2017	49	Septic shock	Mode is unknown, but spontaneous breath is allowed VT = 8–10 ml/kg, PEEP = 5–12 cmH2O	Brachial artery	Classic fluid responsiveness test
LU N [13]	2017	49	Setptic shock	Mode is unknown, but spontaneous breath is allowed VT = 8–10 ml/kg, PEEP = 5–12 cmH2O	Carotid artery	Classic fluid responsiveness test

*VC mode* controlled-volume mode, *PSV mode* pressure support mode, *SIMV mode* synchronized intermittent mandatory ventilation mode, *VT* tidal volume, *PEEP* positive end expiratory pressure, *PPV* pulse pressure variation. Classic fluid responsiveness test: monitor whether cardiac output or stroke volume increases 12, 15% or not after fluid loading

The quality of the included studies was assessed by QUADAS-2 in Table 2. Most studies had no description whether the sample of enrolled patients was consecutive or random. In Zhu W [12] and LU N [13] studies, it was not reported that observers were blinded or did not know the result of golden standard until after monitoring △Vpeak PA. In Roehrig C [6], there were 25 (60%) patients ventilated with synchronized intermittent mandatory ventilation (SIMV) mode and 17 (40%) patients with pressure support mode (PSV) mode. In ZHU W [12] study, all the patients were ventilated with PSV mode. But in LU N [13] study, no details about breath mode were reported. In the rest enrolled studies, the patients were all ventilated with controlled-volume (VC) mode. Classic fluid responsiveness test (monitor whether cardiac output or stroke volume increases 12, 15% or not after fluid loading) is reference standards. But Roehrig C [6] and Brennan JM [7] used PPV and the passive leg raising test separately as the gold standard for predicting fluid responsiveness. Moreover, some patients with stable circulatory status were also included in these two studies [6, 7]. Although classic fluid responsiveness test was performed, vigileo monitor or ultrasound, not thermodilution method, was used to monitor the change of cardiac output in three studies [8–10] (Table 3). Generally, according to QUADAS-2, quality of two studies [6, 7] was low, and these two studies were not included in further analysis.

**Table 2:** Quality assessment of included studies using QUADAS-2

Study	Risk of bias				Applicability concerns		
	Patient selection	Index test	Reference standard	Time and flow	Patient selection	Index test	Reference standard
Roehrig C [6]	●	○	●	○	●	○	●
Brennan JM [7]	●	○	●	○	●	○	●
Monge García MI [8]	●	○	○	○	○	○	●
Yin WH [9]	?	○	○	○	○	○	●
Song Y [10]	●	○	○	○	●	○	●
Ibarra-Estrada MÁ [11]	○	○	○	○	○	○	○
Zhu W [12]	?	?	○	○	○	?	○
LU N [13]^a^	?	?	○	○	○	?	○
LU N [13]^b^	?	?	○	○	○	?	○

○low risk ●high risk? unclear risk

^a^: the peripheral artery is the brachial artery; ^b^:the peripheral artery is the carotid artery

**Table 3:** Details of fluid responsiveness test

Author	Amount of fluid	Type of fluid	Time of infusion	Cardiac output monitor
Monge García MI [8]	500 ml	synthetic colloid	30 min	vigileo monitor
Yin WH [9]	500 ml	6% hydroxyethyl starch 130/0.4	30 min	ultrasound
Song Y [10]	6 ml/kg	6% hydroxyethyl starch 130/0.4	10 min	vigileo monitor
Ibarra-Estrada MÁ [11]	7 ml/kg	normal saline	30 min	PiCCO
Zhu W [12]	500 ml	6% hydroxyethyl starch 130/0.4	30 min	PiCCO
LU N [13]	200 ml	normal saline	10 min	PiCCO

*PICCO* pulse indicator continuous cardiac output

The peripheral artery was the carotid and brachial artery with no radial or femoral artery in the rest of seven included studies. Main diagnostic values of peripheral artery peak velocity variation to predict fluid responsiveness in these seven included studies were shown in Table 4. Because of the different measurement sites (brachial/carotid), there was strong clinical heterogeneity across the included studies. In order to reduce the effect of clinical heterogeneity, the meta-analysis for brachial and carotid sites was performed individually.

**Table 4:** Main diagnostic values of included studies

Author	No. of Responder	No. of Non-responder	AUC value	Cut-off value	Sensitivity	Specificity
*Carotid artery*						
Yin WH [9]	22	24	0.95	12.1%	90.9%	83.3%
Song Y [10]	23	17	0.85	11%	85.0%	82.0%
Ibarra-Estrada MÁ [11]	30	29	0.88	14%	86.0%	86.0%
LU N [13]	27	22	0.91	13%	78.0%	90.0%
*Brachial artery*						
Monge García MI [8]	19	19	0.88	10%	74.0%	95.0%
Zhu W [12]	32	26	0.816	13.3%	71.90%	80.80%
LU N [13]	27	22	0.761	11.7%	70.0%	80.0%

*AUC* area under the receiver operating characteristic curve

**Fig. 2:**
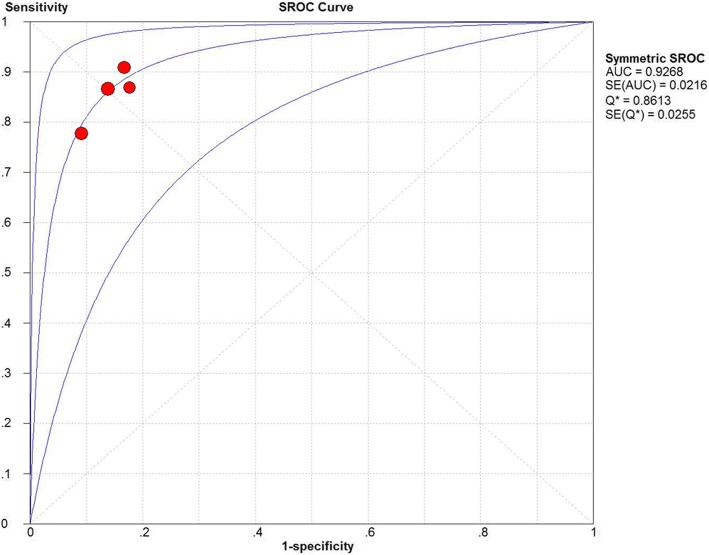
Summary receiver operating characteristic curve of △Vpeak carotid artery in predicting fluid responsiveness

Measurement sites of four studies were carotid artery [9–11, 13]. The Spearman correlation coefficient of sensitivity and 1-specificity logarithmic was 0.800, with no statistical difference (*P* = 0.200), so there was no statistical heterogeneity caused by threshold effect. There was also no significant heterogeneity caused by the non-threshold effect among the included studies; the I^2^ values for sensitivity, specificity, positive likelihood ratio, negative likelihood ratio, and diagnostic odds ratio were 0, 0, 0, 0 and 0% respectively. A fixed-effect model was used for meta-analysis because statistical heterogeneity did not exist. The pooled sensitivity, specificity, positive likelihood ratio, negative likelihood ratio, and diagnostic odds ratio were 0.85 (95% confidence interval (CI) 0.77–0.92), 0.86 (95% CI 0.77–0.92), 6.07 (95% CI 3.64–10.12), 0.17 (95% CI 0.10–0.27), and 38.56 (95% CI 17.05–87.24), respectively. A summary receiver operating characteristic curve yielded an area under the curve of 0.9268, and the Q value was 0.8613 (Fig. 2). Because the sample size of each study was small [15], the Harbord test was applied to determine the presence of publication bias using Stata 14.0 software. The results showed that the publication bias was not found (*P* = 0.666).

**Fig. 3:**
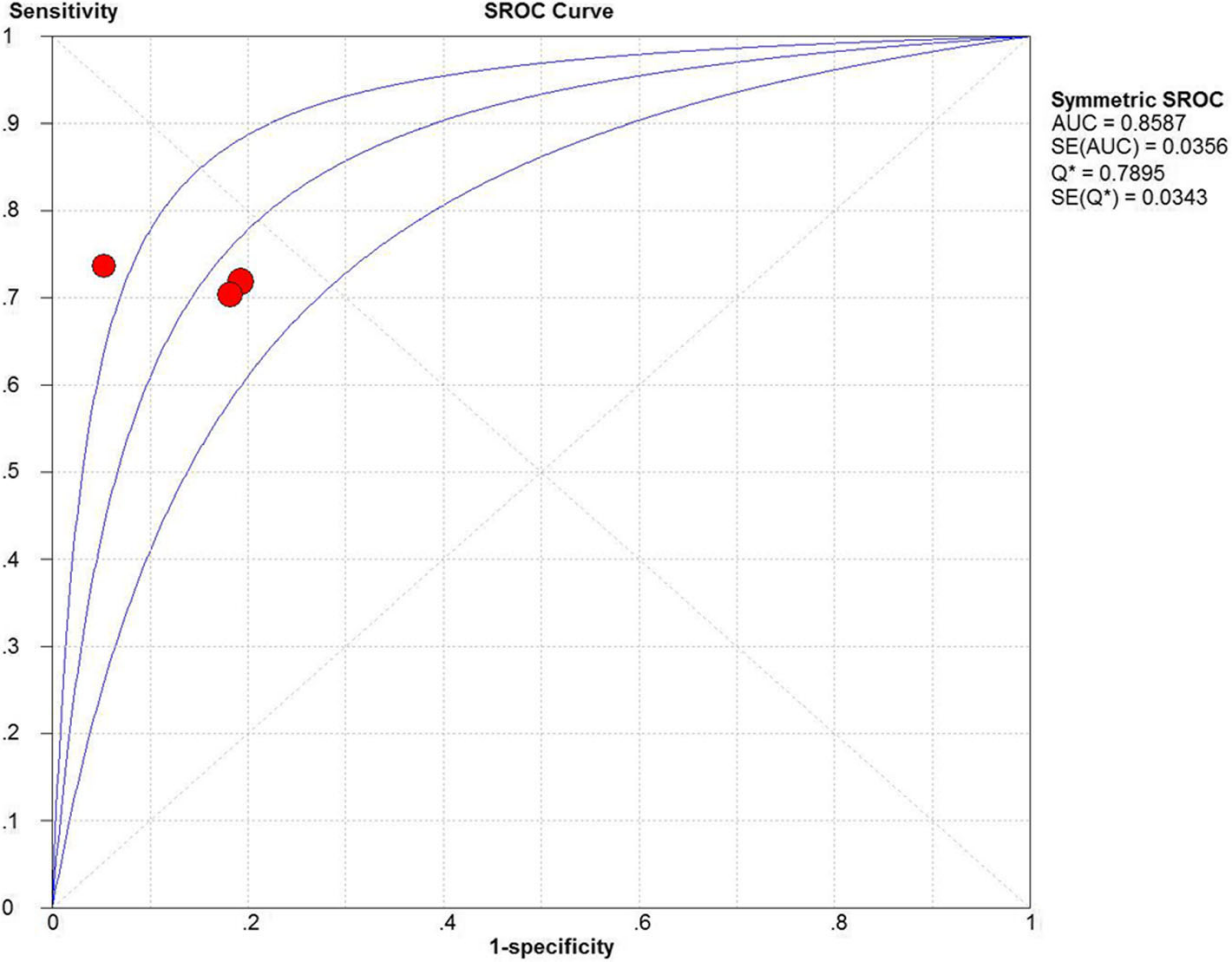
Summary receiver operating characteristic curve of △Vpeak brachial artery in predicting fluid responsiveness

Measurement sites of three studies were brachial artery [8, 12, 13]. The Spearman correlation coefficient of sensitivity and 1-specificity logarithmic was − 0.500, with no statistical difference (*P* = 0.667), so there was no statistical heterogeneity caused by threshold effect. There was also no significant heterogeneity caused by the non-threshold effect among the included studies; the I^2^ values for sensitivity, specificity, positive likelihood ratio, negative likelihood ratio, and diagnostic odds ratio were 0, 13.6, 0, 0 and 0% respectively. A fixed-effect model was used for meta-analysis because statistical heterogeneity did not exist. The pooled sensitivity, specificity, positive likelihood ratio, negative likelihood ratio, and diagnostic odds ratio were 0.72 (95% CI 0.60–0.81), 0.85 (95% CI 0.74–0.93), 4.73 (95% CI 2.64–8.47), 0.33 (95% CI 0.23–0.48), and 14.06 (95% CI 6.26–31.56), respectively. A summary receiver operating characteristic curve yielded an area under the curve of 0.8587, and the Q value was 0.7895 (Fig. 3). Because the sample size of each study was small [15], the Harbord test was applied to determine the presence of publication bias using Stata 14.0 software. The results showed that the publication bias was not found (*P* = 0.263).

## Discussion

In this meta-study, we studied the value of peripheral artery (carotid and brachial artery) peak velocity variation to predict fluid responsiveness in mechanically ventilated patients. Finally, we found that △Vpeak of carotid and brachial artery had a diagnostic value in predicting fluid responsiveness respectively. Moreover, △Vpeak of carotid artery had more value than brachial artery in predicting fluid responsiveness.

In the parameters predicting fluid responsiveness, it was shown that dynamic indicators (SVV and PPV) had more precise diagnostic value than did static indicators (central venous pressure and pulmonary artery wedge pressure) [5]. In a related meta-analysis, the results were similar. In a meta-analysis including 22 studies with 807 patients enrolled, it was reported that PPV was an accurate predictor of fluid responsiveness with a pooled sensitivity of 0.88, a specificity of 0.89, and a summary area of receiver operating characteristic curve of 0.94 [16]. In another meta-analysis including 23 studies with 568 patients enrolled, it was reported that SVV was also an accurate predictor of fluid responsiveness with a pooled sensitivity of 0.81, a specificity of 0.80, and a summary area of receiver operating characteristic curve of 0.93 [17]. In our meta-analysis, it was shown that △Vpeak PA, especially △Vpeak of carotid artery, was also an accurate diagnostic value of fluid responsiveness with a pooled sensitivity of 0.85, a specificity of 0.86, and a summary area of receiver operating characteristic curve of 0.93.

An invasive vessel puncture is needed to obtain an artery pulse wave for calculating PPV or SVV, but we can obtain the △Vpeak PA noninvasively with ultrasound. Although SVV can also be obtained by ultrasound, in 40% of patients, SV at the left ventricle outflow tract was not obtained due to an unclear transthoracic echocardiographic image [11]. In addition, the location of the peripheral artery is shallow enough to obtain a high-quality image. Moreover, it is easy for a beginner in ultrasonography to master the skill of measuring the peripheral arterial blood flow peak velocity. Therefore, it is much easier to obtain the △Vpeak PA parameter than the SVV or PPV parameter.

The fundamental cause of respiratory variation in peripheral arterial blood flow peak velocity was the respiratory variation of stroke volume. From the results of the study, the △Vpeak of carotid artery had more value than brachial artery in predicting fluid responsiveness. The reason may be that carotid artery had the advantage of anatomical location (closer to the heart). Thus, the blood flow of carotid artery is more sensitive to the change of stroke volume than brachial artery. Marik PE found that in volume responders, carotid blood flow increased 79%, but brachial blood flow only increased 12% following fluid loading, that meant there was a preferential distribution of blood towards the carotid circulation and away from the brachial circulation [18].

According to QUADAS-2, two low quality studies were deleted in further statistical analysis in this study. Moreover, because of different locations of peripheral artery, the rest included studies were divided into brachial and carotid sites groups for meta-analysis individually. But in each group, there was also some clinical heterogeneity. Firstly, tidal volume was set higher than 8 ml/kg with no spontaneous respiration in only one study in brachial site group and two studies in carotid site group. If ventilated patients have spontaneous breath or if tidal volume is smaller than 8 ml/kg, the diagnostic value of SVV and PPV would decrease [19]. So, a small tidal volume and spontaneous respiration retained may affect the diagnostic value of △Vpeak PA. Secondly, classic fluid responsiveness test was used. But the method of monitoring cardiac output, amount of fluid, type of fluid and infusion time were not always the same.

In this meta-analysis, non-ventilated patients were excluded. Sheng LF et al. studied the value of brachial peak velocity variation during the Valsalva manoeuvre to predict fluid responsiveness, and their results showed that the area under the receiver operating characteristic curve was 0.903, with a sensitivity of 87% and a specificity of 82% [20]. Similarly, Préau S studied the value of femoral artery peak velocity variation during a deep inspiration manoeuvre to predict fluid responsiveness in spontaneously breathing patients, and they found that it was an accurate index for predicting fluid responsiveness (area under receiver operating characteristic curve of 0.95, sensitivity 95% and specificity of 100%) [21]. However, it is hard for critically ill patients with circulatory failure to cooperate with the commands required for performing the Valsalva manoeuvre or the deep inspiration manoeuvre. It is unfortunate that in quiet spontaneous breath patients, the value of femoral artery peak velocity variation to predict fluid responsiveness decreases (area under the receiver operating characteristic curve of 0.74, sensitivity of 60% and specificity of 100%) [21].

There were some limitations in this meta-analysis. In most of the included studies, patients with arteriostenosis were excluded. Therefore, advanced studies are needed to prove if the results apply to these specific patients. Not every included study showed details for measuring the location of the artery (left or right). Additionally, it was unknown whether there were different results between the left and right arteries. In this meta-analysis, we did not search for the available studies about the value of femoral and radial artery peak velocity variation in predicting fluid responsiveness. Therefore, the peripheral artery in this study only included carotid and brachial arteries. The last but also the biggest limit was that there was some clinical heterogeneity as stated above. The amount of enrolled studies of each group was too small to further analyze the influence of such clinical heterogeneity on the results. So further studies are needed to confirm diagnostic accuracy.

## Conclusion

In the meta-analysis, we evaluated the value of △Vpeak PA to predict fluid responsiveness in mechanically ventilated patients. The results showed △Vpeak of carotid and brachial artery had a diagnostic value in predicting fluid responsiveness respectively. Moreover, △Vpeak of carotid artery had more value than brachial artery in predicting fluid responsiveness. However, there was some clinical heterogeneity; therefore, further studies are needed to confirm diagnostic accuracy.

## Abbreviations

△Vpeak PA: respiratory variation in peripheral arterial blood flow peak velocity
CI: confidence interval
PPV: pulse pressure variation
QUADAS-2: Quality Assessment of Diagnostic Accuracy Studies 2
SVV: stroke volume variation
